# A tennis lesson: sharp practice in the science behind the Sharapova case

**DOI:** 10.1136/postgradmedj-2016-134124

**Published:** 2016-06-01

**Authors:** Arduino Arduini, Victor A Zammit

**Affiliations:** 1 CoreQuest, Manno, Switzerland; 2 Translational and Experimental Medicine, Warwick Medical School, Coventry, UK

**Keywords:** Mildronate, Meldonium, Carnitine, Muscle

Maria Sharapova (and hundreds of other elite athletes) took meldonium, a drug developed at the time of the USSR for the treatment of heart attack and stroke, though it has never been approved for use anywhere outside of the former Soviet Union. Meldonium is an inhibitor of γ-butyrobetaine hydroxylase, an enzyme involved in the carnitine biosynthetic pathway.^1^ Intake results in a reduction of tissue carnitine content, including the heart and skeletal muscles. Carnitine plays a critical role in transferring long-chain fatty acids across mitochondrial inner membrane into the mitochondrial matrix, to enable entry of the fatty acid moiety into the oxidation pathway, to synthesise ATP aerobically.^2^ In cases when intracellular carnitine availability is compromised, such as in neonatal primary carnitine deficiency,^3^ those affected experience hypoglycaemia, fatigue, seizures and cardiomyopathy.^3^ Carnitine-deficient individuals are sensitive to prodrugs containing the pivaloyl moiety, as pivalic acid is a prodrug able to induce carnitine deficiency (like meldonium); this results in lethal cardiac arrhythmias.^4^ Meldonium administered orally at ‘recommended’ doses (500 mg, twice daily)^5^ would not have such a drastic effect. However, even a moderate reduction of muscle carnitine content in healthy volunteers treated with pivaloyl-conjugated antibiotics for 54 days reduces work at maximal oxygen uptake (VO_2_max), maximal heart rate,^6^ reduced interventricular septum thickness and left ventricular mass.^7^ The oral administration of meldonium to healthy volunteers (500 mg, twice daily) for 4 weeks leads to a significant 18% reduction of plasma carnitine.^5^ Even though we are not aware of the meldonium dosage prescribed to Ms Sharapova, it is safe to assume that, in the absence of oral carnitine supplementation, her muscle carnitine content would have been, at most, only modestly decreased, and would have resulted in a minimal impact on her muscle oxidative capacity, as evidenced by her continued performance at the highest levels in her sport. The medical team looking after Ms Sharapova appears to have confounded the tangible beneficial effects of depressing acylcarnitine formation in *ischaemic* heart (which may improve glucose use, eg, during ischaemia–reperfusion) with the requirement of normal cardiac muscle for high rates of carnitine-dependent fatty acid oxidation. A recent study in rodents has shown that even a moderate, silent carnitine-deficient phenotype may reveal its fragility when subjected to strong adrenergic stress.^8^ Therefore, unless meldonium has unidentified off-target pharmacological effects, one has to be thankful that it was not administered in high enough doses so as to damage the athletes who continued to take it.

Another aspect of meldonium pharmacology is the inhibition of carnitine acetyltransferase (CAT) activity,^9^ an enzyme that reversibly transfers the acetyl moiety of acetyl coenzyme A to carnitine in mitochondria and peroxisomes. Due to its capacity to buffer mitochondrial acetyl coenzyme A levels, CAT plays a prominent role in intermediary metabolism and muscle bioenergetics.^2^ As expected, in rodent models, a lowered muscle CAT activity results in muscle fatigue and exercise intolerance.^10^ This would again strongly argue against the use of meldonium as a performance-enhancing drug, particularly if such CAT-inhibitory activity is combined with carnitine depletion.

The World Anti-Doping Agency (WADA) may have decided to add meldonium to the Prohibited List, not necessarily because of any illusory performance-enhancing action in healthy athletes, but because of the real danger that it carries for the overenthusiastic athlete who may overdose on it and lower intracellular carnitine to pathological levels. A comprehensive literature search of several databases failed to unearth any cogent evidence of the potential meldonium effect in enhancing physical performance in healthy subjects, with only one clinical trial conducted on a small cohort of old (average >60 year) patients with *angina pectoris*, to evaluate exercise tolerance. On such minimal findings is the dangerous extrapolation to an assumed performance-enhancing effect of meldonium in healthy elite athletes based. It is not an overstatement to emphasise that the understanding of metabolism is a postmodernist discipline in which neither the inadequately trained scientists (Metabolism is a Cinderella subject in most universities) nor the editors of journals or the clinicians who peruse their publications know how to interpret the intricacies of control in metabolism. This is an era in which metabolic science is overrun by overemphasis on gross changes in gene expression, and too little on the reductionist discipline of the study of the function of proteins, their interactions, and their effects on metabolic fluxes. Athletes chasing the shadows of marginal gains are the unwitting victims. Hats off to WADA who may have recognised that, even in the absence of any performance-enhancing properties of meldonium, they should ban it for health reasons. If anything, Ms Sharapova and other ill-advised athletes may not have attained their true athletic potential as a result of having taken meldonium.
